# Polyelectrolyte-Stabilised Magnetic-Plasmonic Nanocomposites

**DOI:** 10.3390/nano8121044

**Published:** 2018-12-13

**Authors:** Shelley Stafford, Coralie Garnier, Yurii K. Gun’ko

**Affiliations:** 1 School of Chemistry, Trinity College Dublin, Dublin 2, Ireland; sstaffo@tcd.ie; 2 Institute of Chemistry of Clermont-Ferrand, Sigma Clermont, 63170 Aubiere, France; coralie.garnier@sigma-clermont.fr; 3 Information Optical Technology Centre, ITMO University, 197101 Saint Petersburg, Russia

**Keywords:** magnetic, plasmonic, nanoparticles, polyelectrolyte, multimodal

## Abstract

In this work, new magnetic-plasmonic nanocomposites have been developed through the use of two complementary polyelectrolytes–polystyrene sulfonate (PSS) and poly(allylamine hydrochloride) (PAH). PSS, a negatively charged polyelectrolyte, was utilized as a stabiliser for magnetite nanoparticles, and PAH, a positively charged polyelectrolyte, was used to stabilize gold nanoparticles. The combination of these two entities resulted in a magnetic-plasmonic nanocomposite that is highly reproducible and scalable. This approach was found to work for a variety of PSS concentrations. The produced magnetic-plasmonic nanomaterials have been characterized by vibrational sample magnetometry (VSM), transmission electron microscopy (TEM) and UV-Vis spectroscopy. These nanocomposite materials have the potential to be used in a variety of biological applications including bioseparation and biosensing.

## 1. Introduction

In recent years, magnetic-plasmonic nanostructures have been the subject of extensive research due to their vast number of potential applications, including magnetic hyperthermia, photothermal therapy, sensing, imaging and theranostics [1,2,3,4]. A wide variety of core-shell magnetic-plasmonic structures have been reported: primarily, these consist of magnetic core-plasmonic shell structures. While this is the most commonly utilised approach to the synthesis of magnetic-plasmonic nanomaterials, within it lie a number of inherent difficulties. Any addition of a non-magnetic material to the surface of magnetic nanoparticles will have a significant impact on the magnetic moment, hence limiting the potential applications. Reproducibility is a key issue amongst core-shell type structures, with incredibly precise synthetic procedures and reaction conditions that must be adhered to in order to prevent separate seeding and subsequent growth of separate plasmonic nanoparticles. Direct coating of a plasmonic layer onto a magnetic core, or vice versa, is incredibly difficult due to the mismatch in their respective crystal lattices [5]. Often, the use of intermediate layers such as silica, polypyrrole (PPY) and polyethylenemine (PEI) have been used to assist in the seeding of plasmonic nanoparticles onto the surface of a magnetic core, thus allowing for their subsequent reduction [6,7,8]. However, this approach will also contribute to the reduction of the magnetic moment of the nanocomposite. It has also been previously demonstrated that magnetic and plasmonic species can be linked using bifunctional molecular linkers [9]. 

The combination of magnetic and plasmonic moieties is the basis for many new multifunctional materials with unique multimodal properties. The primary goal in synthesising such materials is to retain the full functionality of both the magnetic and plasmonic components. Magnetic nanoparticles have long been a material of interest due to their use as contrast agents in magnetic resonance imaging (MRI), magnetic fluids and data storage components [10,11]. When magnetite nanoparticles are below 10 nm in size, they exist as a single domain and are superparamagnetic [12]. This is crucial for many biomedical applications as the nanoparticles have no retention of the magnetic field and hence will not aggregate in vivo. Thus, magnetite nanoparticles are prime candidates for applications such as MRI, magnetic hyperthermia, and magnetically guided drug delivery, as they are also biodegradable, biocompatible, easy to synthesise and have a high magnetic moment [13]. Plasmonic nanoparticles, such as gold, are popular due to its surface plasmon resonance (SPR), thermal and chemical stability, simple surface functionalisation and high electrical and thermal conductivity. Due to the SPR, gold nanoparticles also have a large extinction cross section and local electromagnetic field amplification [14]. The combination of magnetic and plasmonic modalities in one material enables the demonstration of new unique effects such as plasmon induced magneto-optical enhancement [15,16] and magnetic nanoparticle enhanced surface plasmon resonance sensing [17], opening a range of new potential applications [4,18].

A wide variety of approaches have been utilised to coat gold onto magnetite and vice versa. Silica is often used as an intermediate media between the two components, acting as a surface for seeding of the gold nanoparticles and facilitating further functionalisation [6]. A range of polymers, such as polypyrrole (PPY), polyvinylpyrrolidone (PVP, and polyethylineimnine (PEI) have also been used to form charged shells around the central magnetic core, facilitating the seeding and growth of a plasmonic layer [7,8,19]. An alternative approach to a magnetic-plasmonic nanocomposite was described by Ivashchenko et al. who prepared self-organising silver and small iron oxide nanoparticles using a green synthetic approach [20]. In this work, ginger rhizome extract was used as a stabilising agent and the resulting compound was an Ag-Fe_3_O_4_ nanocomposite matrix. In our work, we have developed new magnetic-plasmonic nanocomposites bound through electrostatic interactions. PAH (polyallylamine hydrochloride) and PSS (polystyrene-4-sulfonate) were chosen as the positive and negatively charged polyelectrolytes, respectively, as they are both non-toxic, biocompatible and already have found uses in biomedicine [21,22]. To the best of our knowledge, this is the first report of a magnetic-plasmonic nanocomposite material bound entirely through the electrostatic interactions of oppositely charged polyelectrolytes. 

## 2. Materials and Methods

Iron (III) chloride hexahydrate (>98%), Iron (II) chloride tetrahydrate (99.99%), gold (III) chloride trihydrate (99.9%), ammonium hydroxide solution (99.99%), sodium borohydride (>98%) and poly(allylamine hydrochloride) (M.W. 56,000) and acetic acid (>99.99%) were all purchased from Sigma Aldrich (Dublin, Ireland). Poly(styrene sulfonic acid) (M.W. 300,000) was purchased from Alfa Aesar (Dublin, Ireland).

### 2.1. Synthesis of PSS Stabilised Magnetite Nanoparticles

PSS solutions were made up to the concentrations shown in Table 1. Using degassed Millipore water, an iron solution (0.06 M) was prepared using FeCl_3_·6H_2_O (1.1 g, 4 mmol) and FeCl_2_·4H_2_O in degassed Millipore water. PSS solution (30 mL, concentrations A–D) was then added to a round bottom flask (RBF). The amount of iron solution was varied in order to produce nanocomposites with different ratios of PSS/Magnetite (Table 1)

The solution was stirred under argon at 90 °C for 15 min. The pH was adjusted to 9 using NH_4_OH and the precipitated nanoparticles were stirred at 90 °C for 60 min. The nanoparticles were isolated using magnetic separation and washed with Millipore water (5 × 20 mL).

### 2.2. Synthesis of PAH Stabilised Gold Nanoparticles

Chloroauric acid (0.1 M) and sodium borohydride (0.1 M) solutions were prepared using degassed Millipore water. PAH (0.025 g, mw = 56,000) was added to Millipore water in a 15 mL RBF and degassed with Argon. Chloroauric acid solution (200 μL) was added to the RBF and stirred for 15 min. Sodium borohydride (400 μL) was added dropwise to the gold solution and left to stir for 45 min. A dark red solution indicated the formation of gold nanoparticles. The gold nanoparticles were isolated through centrifugation and washed with methanol (5 × 20 mL).

### 2.3. Synthesis of Gold Nanoparticles (Unstabilised)

Gold nanoparticles were prepared according to a previously reported procedure [23]. Briefly, Millipore water (10 mL) was added to a RBF. Chloroauric acid (200 μL) was added and the solution was stirred for 5 min. Sodium borohydride (400 μL) was added dropwise, and the resulting solution was left to stir for 45 mins. The nanoparticles were isolated through centrifugation and washed with methanol (5 × 20 mL).

### 2.4. Synthesis of Acetic-Acid Stabilised Gold Nanoparticles

PSS-stabilised Fe_3_O_4_ nanoparticles (30 mg) were dispersed in Millipore water (50 mL) and sonicated for 15 mins. This solution was then degassed using Argon. (x) μL of gold (III) chloride solution (0.1 M) in (y) μL of acetic acid was then added. 

### 2.5. Synthesis of Magnetic Plasmonic Nanocomposites

The formation of the magnetic plasmonic nanocomposites was achieved through monitoring using UV-Vis. In each case, the gold nanoparticle solution was added until the plasmon peak was clearly visible (approximately a.u. 0.5). Following this, the nanocomposite was isolated using magnetic separation and washed 5 times before analysis was carried out. 

## 3. Results and Discussion

### 3.1. Synthesis of Magnetic-Plasmonic Nanocomposites

The layer-by-layer assembly technique based on electrostatic interactions is widely used for the preparation of nanoscale films on various substrates for a range of applications [9,24,25,26,27,28]. In our approach, we used polyelectrolyte mediated electrostatic interactions between gold and magnetite nanoparticles for the assembly of new magnetic-plasmonic nanocomposites in solution.

**Figure 1:**
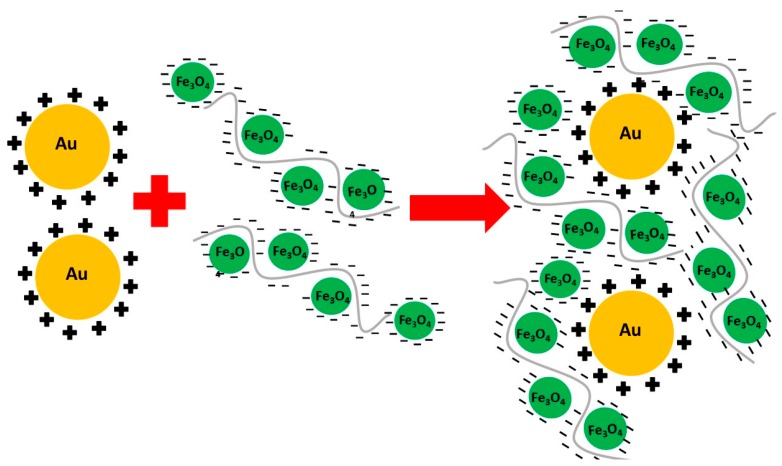
Scheme illustrating the formation of the magnetic-plasmonic nanocomposite. The minus signs around the Fe_3_O_4_ nanoparticles indicate the negative charge of the PSS stabiliser, and the positive signs around the Au nanoparticles indicate the positive charge of the PAH stabiliser.

The PAH-stabilised gold nanoparticle solution was added sequentially to each concentration of PSS-stabilised magnetite until a strong plasmon peak was obtained. A scheme illustrating the interactions of the polyelectrolyte stabilized magnetite and gold nanoparticles is shown in Figure 1. The magnetite nanoparticles are crosslinked by the PSS stabilizer, and electrostatically bound to the PAH-stabilised gold nanoparticles, embedding them within a composite matrix. 

The produced magnetic-plasmonic nanocomposites have been characterized by vibrational sample magnetometry (VSM), transmission electron microscopy (TEM) and UV-Vis spectroscopy.

### 3.2. VSM Analysis of Nanocomposites

**Figure 2:**
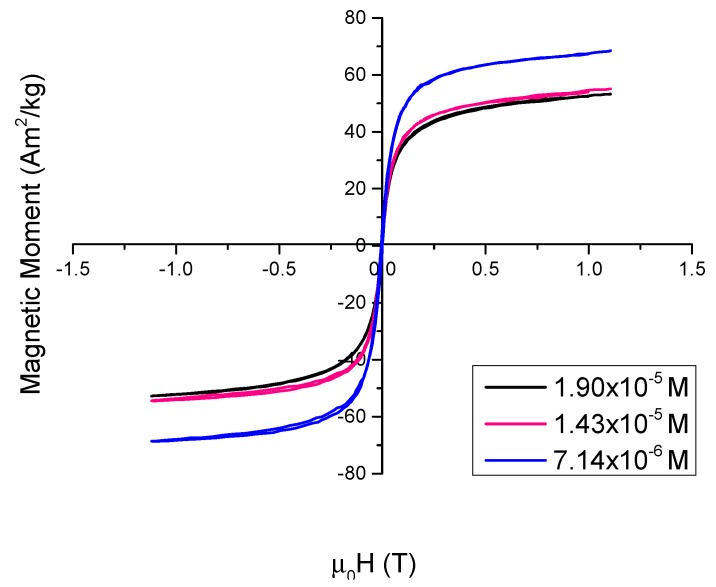
VSM (Vibrating Sample Magnetometry) curves of each concentration of immobilized PSS-stabilised Fe_3_O_4_ nanoparticles.

VSM analysis was performed at room temperature (295 K). The absence of a hysteresis loop confirms that these nanoparticles display superparamagnetic behaviour at room temperature (Figure 2). Lower concentrations of PSS result in magnetite nanoparticles that have higher saturation magnetisations: 53.2 Am^2^/kg for 1.90 × 10^−5^ M, 55.1 Am^2^/kg for 1.43 × 10^−5^ M and 68.5 Am^2^/kg for 7.14 × 10^−6^ M. This was expected as the polyelectrolyte itself is not magnetic and thus increasing concentrations of PSS would reduce the effective magnetic moment of the magnetite. Higher concentrations of PSS decrease the magnetization due to the fact that PSS is a non-magnetic material but adds to the total sample mass. These figures are comparable to the saturation value for bulk magnetite ~90 Am^2^/Kg. 

### 3.3. TEM and UV-Vis Analysis of Nanocomposites

**Figure 3:**
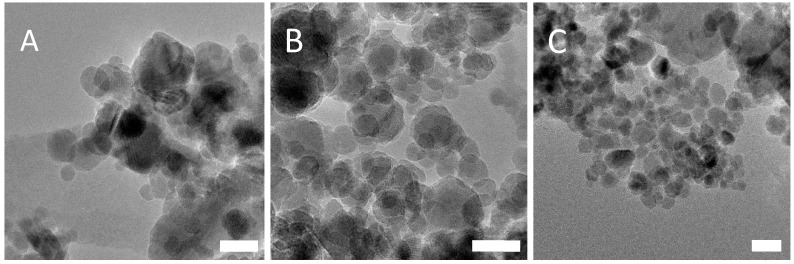
TEM images of PSS-stabilised Fe_3_O_4_ for each concentration of PSS (**A**) 1.90 × 10^−5^ M, (**B**) 1.43 × 10^−5^ M and (**C**) 7.14 × 10^−6^ M. Scale bars correspond to 20 nm.

TEM analysis for PSS-stabilised magnetite found no major differences between nanoparticle diameters for each concentration of PSS tested (Figure 3). Size distribution analysis found that the average size of the nanoparticles was 10.8 nm ± 3.3 nm for 1.90 × 10^−5^ M, 10.3 nm ± 2.6 nm for 1.43 × 10^−5^ M and 10.5 nm ± 3.2 nm for 7.14 × 10^−6^ M. Size distribution analysis may be found in the Supplementary Materials (S1). Each concentration of PSS stabilized Fe_3_O_4_ nanoparticles was then tested with three different gold nanoparticle samples—unstabilised gold, acetic acid stabilized gold and PAH-stabilised gold. This was carried out to determine if the electrostatic interaction between the two polyelectrolytes is an effective method of binding the Fe_3_O_4_ and gold nanoparticle species, and also to demonstrate that, in the absence of the PAH or even in the presence of other stabilisers, the gold retention through multiple magnetic separations would not take place. DLS (Dynamic Light Scattering) analysis of the PSS-stabilised Fe_3_O_4_ nanocomposites can be found in the Supplementary Materials (S2). 

**Figure 4:**
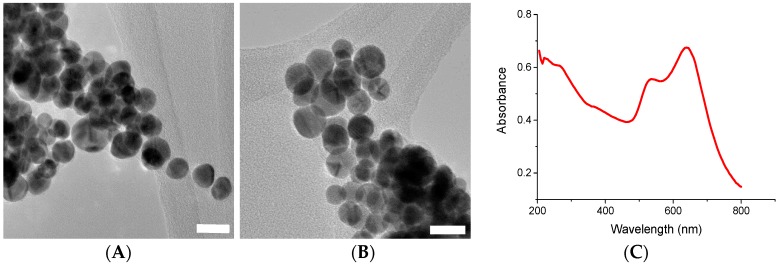
(**A**) and (**B**) are TEM images of PAH-stabilised Au nanoparticles. The scale bars correspond to 20 nm. (**C**) shows the UV-Vis spectrum for the PAH-stabilised gold nanoparticles.

UV-Vis analysis of PAH-stabilised gold nanoparticles showed two plasmon peaks, at 530 nm and 640 nm, respectively (Figure 4C). Upon TEM analysis, it was determined the presence of these two peaks was due to two slightly different size distributions of gold nanoparticles, and not due to the presence of non-spherical particles such as nanorods, as one might suspect from such a system (Figure 4A,B). The gold nanoparticles have a mean size of 13.7 nm ± 3.5 nm. Size distribution plots for PAH-stabilised gold nanoparticles can be found in the Supplementary Materials (S3). The two plasmon peaks are thought to be due to the polyelectrolyte stabilizer crosslinking the particles and causing the formation of larger clusters [19]. DLS analysis of the sample confirmed this theory. DLS analysis can be found in the Supplementary Materials (S4).

**Figure 5:**
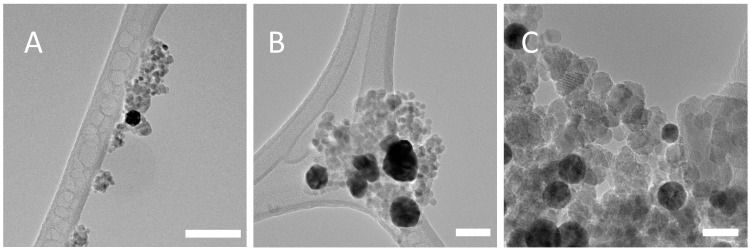
TEM images of PSS-Stabilised Magnetite and Unstabilised Au nanoparticles, (**A**) PSS concentration 1.90 × 10^−5^ M, scale bar = 100 nm; (**B**) PSS concentration 1.43 × 10^−5^ M, scale Bar = 50 nm; (**C**) PSS concentration 7.14 × 10^−6^ M, scale bar = 20 nm. The large, dark nanoparticles are gold and the smaller lighter grey particles are magnetite.

Representative TEM images of the PSS-stabilised Fe_3_O_4_ and unstabilised gold nanoparticles are shown in Figure 5. The gold nanoparticles synthesized in this manner were considerably larger, in the region of 50 nm in diameter; this is due to the lack of stabilizer on the gold nanoparticles and so no species is present to prevent gold growth. After five magnetic separations, the concentration of Au NPs present is low, which is as expected as there is no electrostatic interaction between the PSS-stabilised Fe_3_O_4_ and the unstabilised Au nanoparticles, and so the gold nanoparticles are lost through successive magnetic separations and washings. This was confirmed through UV-Vis analysis on the washings, which showed no gold plasmon peaks.

**Figure 6:**
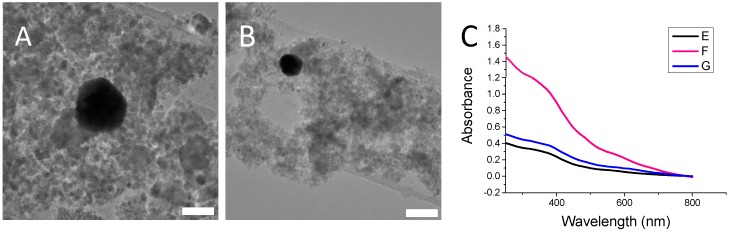
(**A**) TEM images of PSS-Stabilised Fe_3_O_4_ and acetic acid stabilized Au NPs. Scale bar = 50 nm; (**B**) TEM image of PSS-Stabilised Fe_3_O_4_ and acetic acid stabilized Au NPs. Scale bar = 100 nm; (**C**) UV-Vis spectra for each acetic acid stabilized Au and PSS-stabilised Fe_3_O_4_ nanocomposite after multiple magnetic separations. E, F and G refer to the three samples of acetic acid stabilized gold nanoparticles that were prepared with varying amounts of gold (III) chloride and acetic acid solutions. These can be found in Table 2.

Representative TEM images and UV-Vis spectra for the PSS-stabilised Fe_3_O_4_ and acetic acid stabilized gold nanocomposite are shown in Figure 6. Similar to the case for the unstabilised gold nanoparticles, a very low concentration of gold was present for each concentration of PSS (two representative TEMs are shown here). The gold concentration is sufficiently low in the nanocomposite after five successive magnetic washings that a plasmon peak is no longer visible in the UV-Vis spectrum for each concentration of acetic acid stabilized gold that was tested. 

**Figure 7:**
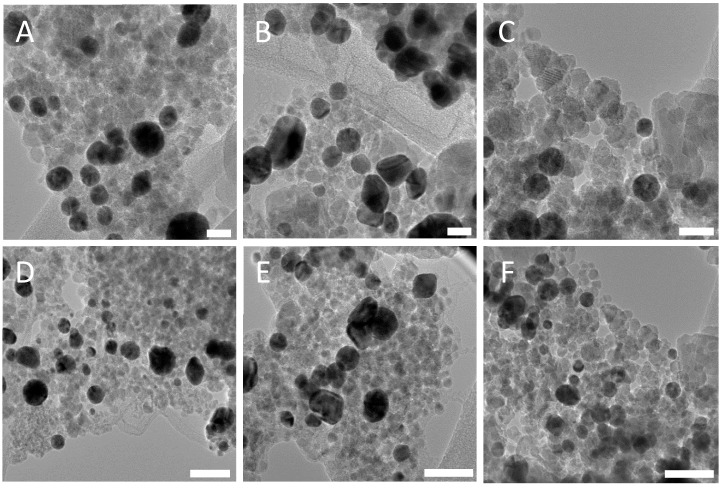
TEM images of PSS-stabilised Fe_3_O_4_ and PAH-stabilised Au nanoparticles. Scale bars are 20 nm in (**A**–**D**) and 50 nm in (**E**,**F**). (**A**,**D**) are 1.90 × 10^−5^ M PSS-stabilised Fe_3_O_4_ and gold nanoparticles, (**B**,**E**) are 1.43 × 10^−5^ M PSS-stabilised Fe_3_O_4_ nanoparticles and gold nanoparticles; and (**C**,**F**) are 7.14 × 10^-6^ M PSS-stabilised Fe_3_O_4_ nanoparticles and gold nanoparticles.

**Figure 8:**
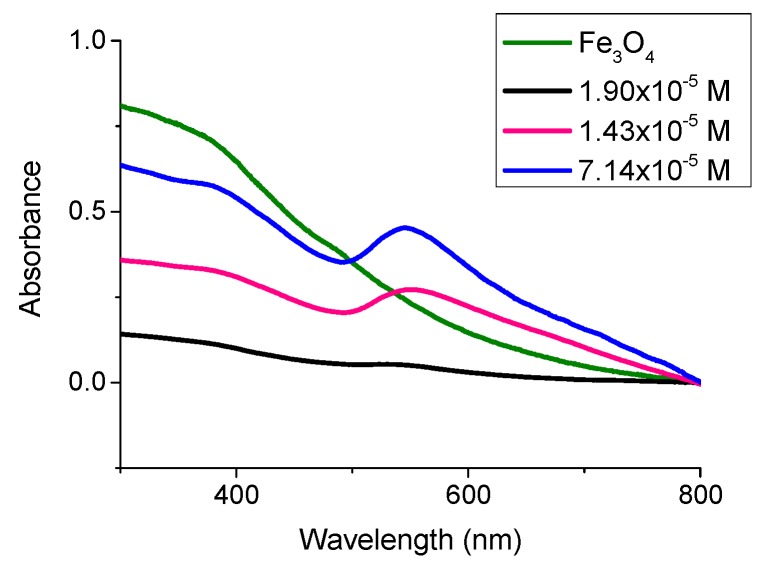
UV-Vis spectra of each PSS-Magnetite concentration titrated with PAH-stabilised gold after five magnetic separations.

TEM analysis of the PSS-stabilised Fe_3_O_4_ and PAH-stabilised Au nanocomposite showed a large retention of the gold for each concentration of PSS after multiple magnetic separations (Figure 7). This demonstrates that the electrostatic interactions between the two oppositely charged polyelectrolytes stabilizing the Fe_3_O_4_ and gold nanoparticles is sufficient to hold the two species together. This was further confirmed by UV-Vis analysis (Figure 8). A strong plasmon peak is still clear after five successive magnetic separations in PSS concentrations 1.43 × 10^−5^ M and 7.14 × 10^−6^ M. It is thought that the concentration of magnetite in the PSS 1.90 × 10^−5^ M sample is high enough that it is obscuring the plasmon peak in the UV-Vis, as TEM analysis of the same sample reveals a high concentration of PAH-stabilised gold in the nanocomposite. It is clear that the addition of Au nanoparticles to PSS-stabilised magnetite with concentrations larger than 1.43 × 10^−5^ M allows for the presence of plasmonic resonance, as at the large excess of Au nanoparticles they are not affected by magnetisation value and can retain their original plasmonic properties. The gold nanoparticles are significantly larger than the magnetite nanoparticles in the nanocomposite dispersion; however, this does not seem to have any significant impact on the formation of the nanocomposite. As long as the size differences are not so large as to cause one of the species to precipitate out of solution, the electrostatic interaction between the two species will still take place. Zeta potential measurements for PSS-stabilised Fe_3_O_4_ nanoparticles and PAH-stabilised gold nanoparticles have shown values of −17.2 mV and +19.1 mV, respectively (see Figures S5 and S6 in Supplementary Materials).

## 4. Conclusions

Thus, a new magnetic-plasmonic nanocomposite consisting of PSS-stabilised Fe_3_O_4_ and PAH-stabilised Au nanoparticles has been prepared through a simple, highly reproducible coprecipitation method. The gold is retained in the solution through multiple magnetic separations. A variety of initial PSS concentrations and precursor iron ratios were tested with a single concentration of PAH-stabilised gold nanoparticles. Unstabilised gold nanoparticles and gold nanoparticles stabilised with acetic acid were also prepared and tested with the PSS-stabilised magnetite. It was found in both cases and for each concentration that the gold nanoparticles were lost through the magnetic separation process. This is further proof that it is the electrostatic interactions between the oppositely charged polyelectrolytes stabilising the magnetite and gold nanoparticles that causes the retention of the gold nanoparticles through multiple magnetic washings. For each concentration of PSS that was tested, retention of the gold nanoparticles was attained through multiple magnetic separations.

To the best of our knowledge, this is the first magnetic-plasmonic nanocomposite with components completely bound through electrostatic interactions. The use of this approach enabled us to completely avoid many of the difficulties encountered when preparing core-shell magnetic-plasmonic nanocomposites, such as the need for intermediate layers due to the incompatibility of the magnetic ferrites and noble metals, and reproducibility issues that arise from separate seeding of nanoparticles and preferential growth of separate species rather than seeding onto the core nanoparticle. 

The binding effectiveness of the two complementary polyelectrolyte stabilisers after multiple magnetic separations was demonstrated through testing the same method using unstabilised- and acetic acid-stabilized gold nanoparticles. It was found in each of these cases that the majority of the gold that was initially present in the nanocomposite was removed during the magnetic separations. This is not seen with the polyelectrolyte nanocomposite system, which shows a large retention of gold nanoparticles after five magnetic separations, and also works over a range of PSS concentrations. 

We believe that this approach could be extended to the preparation of other polyelectrolyte stabilized nanoparticulate composites, as well as other magnetic ferrite/plasmonic nanoparticles systems, depending on the targeted application. Future work with these nanocomposites will include further functionalization of the gold nanoparticles with a targeting moiety and testing its separation and sensing capabilities for toxins and heavy metals in water.

## Figures and Tables

**Table 1:** PSS (polystyrene-4-sulfonate) concentrations and iron precursor ratios used to obtain each PSS-stabilised magnetite concentration.

Solution	PSS Concentration	Iron Precursor Ratio
A	1.90 × 10^−5^ M	1384
B	1.43 × 10^−5^ M	1800
C	7.14 × 10^−5^ M	3614

**Table 2:** Amounts of gold (III) chloride solution (x (μL)) and acetic acid (y (μL)) used to synthesise acetic acid stabilized gold nanoparticles.

	x (μL)	y(μL)
E	300	1.5
F	600	3
G	2400	12

## Supplementary Materials

The following are available online at http://www.mdpi.com/2079-4991/8/12/1044/s1, Figure S1: Size distribution analysis for PSS-stabilised magnetite, Figure S2: DLS analysis of PSS-stabilised Fe_3_O_4_ nanoparticles, Figure S3: Size distribution analysis for PAH-stabilised gold nanoparticles, Figure S4: DLS analysis of PAH-stabilised gold nanoparticles. Figure S5: Zeta potential measurements for PSS-stabilised Fe_3_O_4_ nanoparticles. Figure S6: Zeta potential measurements for PAH-stabilised gold nanoparticles.

## References

[1] Lai J.-J., Lai W.-R., Chen C.-Y., Chen S.-W., Chiang C.-L. (2013). Multifunctional magnetic plasmonic nanoparticles for applications of magnetic/photo-thermal hyperthermia and surface enhanced Raman spectroscopy. J. Magn. Magn. Mater..

[2] Pastoriza-Santos I., Kinnear C., Pérez-Juste J., Mulvaney P., Liz-Marzán L.M. (2018). Plasmonic polymer nanocomposites. Nat. Rev. Mater..

[3] Ravichandran M., Oza G., Velumani S., Ramirez J.T., Garcia-Sierra F., Andrade N.B., Vera A., Leija L., Garza-Navarro M.A. (2016). Plasmonic/magnetic multifunctional nanoplatform for cancer theranostics. Sci. Rep..

[4] Stafford S., Serrano Garcia R., Gun’ko Y. (2018). Multimodal magnetic-plasmonic nanoparticles for biomedical applications. Appl. Sci..

[5] Jana N.R. (2011). Design and development of quantum dots and other nanoparticles based cellular imaging probe. Phys. Chem. Chem. Phys..

[6] Jin X., Li H., Wang S., Kong N., Xu H., Fu Q., Gu H., Ye J. (2014). Multifunctional superparamagnetic nanoshells: Combining two-photon luminescence imaging, surface-enhanced raman scattering and magnetic separation. Nanoscale.

[7] He C., Nie W., Feng W. (2014). Engineering of biomimetic nanofibrous matrices for drug delivery and tissue engineering. J. Mater. Chem. B.

[8] Vaitkuviene A., Kaseta V., Voronovic J., Ramanauskaite G., Biziuleviciene G., Ramanaviciene A., Ramanavicius A. (2013). Evaluation of cytotoxicity of polypyrrole nanoparticles synthesized by oxidative polymerization. J. Hazard. Mater..

[9] Brullot W., Strobbe R., Bynens M., Bloemen M., Demeyer P.-J., Vanderlinden W., De Feyter S., Valev V.K., Verbiest T. (2014). Layer-by-layer synthesis and tunable optical properties of hybrid magnetic–plasmonic nanocomposites using short bifunctional molecular linkers. Mater. Lett..

[10] Stephen Z.R., Kievit F.M., Zhang M. (2011). Magnetite nanoparticles for medical mr imaging. Mater. Today (Kidlington Engl.).

[11] Tietze R., Zaloga J., Unterweger H., Lyer S., Friedrich R.P., Janko C., Pöttler M., Dürr S., Alexiou C. (2015). Magnetic nanoparticle-based drug delivery for cancer therapy. Biochem. Biophys. Res. Commun..

[12] Wu K., Wang J.-P. (2017). Magnetic hyperthermia performance of magnetite nanoparticle assemblies under different driving fields. AIP Adv..

[13] Sun C., Lee J.S.H., Zhang M. (2008). Magnetic nanoparticles in mr imaging and drug delivery. Adv. Drug Deliv. Rev..

[14] Amendola V. (2016). Surface plasmon resonance of silver and gold nanoparticles in the proximity of graphene studied using the discrete dipole approximation method. Phys. Chem. Chem. Phys..

[15] López-Ortega A., Takahashi M., Maenosono S., Vavassori P. (2018). Plasmon induced magneto-optical enhancement in metallic ag/feco core/shell nanoparticles synthesized by colloidal chemistry. Nanoscale.

[16] Wang L., Clavero C., Huba Z., Carroll K.J., Carpenter E.E., Gu D., Lukaszew R.A. (2011). Plasmonics and enhanced magneto-optics in core−shell co−ag nanoparticles. Nano Lett..

[17] Wang J., Munir A., Zhu Z., Zhou H.S. (2010). Magnetic nanoparticle enhanced surface plasmon resonance sensing and its application for the ultrasensitive detection of magnetic nanoparticle-enriched small molecules. Anal. Chem..

[18] Fan Z., Shelton M., Singh A.K., Senapati D., Khan S.A., Ray P.C. (2012). Multifunctional plasmonic shell–magnetic core nanoparticles for targeted diagnostics, isolation, and photothermal destruction of tumor cells. ACS Nano.

[19] Le Garrec D., Gori S., Luo L., Lessard D., Smith D.C., Yessine M.A., Ranger M., Leroux J.C. (2004). Poly(n-vinylpyrrolidone)-block-poly(d,l-lactide) as a new polymeric solubilizer for hydrophobic anticancer drugs: In vitro and in vivo evaluation. J. Control. Release.

[20] Ivashchenko O., Gapiński J., Peplińska B., Przysiecka Ł., Zalewski T., Nowaczyk G., Jarek M., Marcinkowska-Gapińska A., Jurga S. (2017). Self-organizing silver and ultrasmall iron oxide nanoparticles prepared with ginger rhizome extract: Characterization, biomedical potential and microstructure analysis of hydrocolloids. Mater. Des..

[21] Georgianos P.I., Liampas I., Kyriakou A., Vaios V., Raptis V., Savvidis N., Sioulis A., Liakopoulos V., Balaskas E.V., Zebekakis P.E. (2017). Evaluation of the tolerability and efficacy of sodium polystyrene sulfonate for long-term management of hyperkalemia in patients with chronic kidney disease. Int. Urol. Nephrol..

[22] Janeesh P.A., Sami H., Dhanya C.R., Sivakumar S., Abraham A. (2014). Biocompatibility and genotoxicity studies of polyallylamine hydrochloride nanocapsules in rats. RSC Adv..

[23] Campos W.N.D.S., Leite A.E.T., Sonego D.A., Andrade M.A.D., Pizzinatto F.D., Marangoni V.S., Zucolotto V., Nakazato L., Colodel E.M., Souza R.L.D. (2017). Síntese e caracterização de nanopartículas de ouro conjugadas com curcumina e seus efeitos na osteoartrite experimental induzida. Ciência Rural.

[24] Srivastava S., Kotov N.A. (2008). Composite layer-by-layer (lbl) assembly with inorganic nanoparticles and nanowires. Acc. Chem. Res..

[25] Chapel J.P., Berret J.F. (2012). Versatile electrostatic assembly of nanoparticles and polyelectrolytes: Coating, clustering and layer-by-layer processes. Curr. Opin. Colloid Interface Sci..

[26] Borges J., Mano J.F. (2014). Molecular interactions driving the layer-by-layer assembly of multilayers. Chem. Rev..

[27] Richardson J.J., Cui J.W., Bjornmalm M., Braunger J.A., Ejima H., Caruso F. (2016). Innovation in layer-by-layer assembly. Chem. Rev..

[28] Hu Z.C., Huang F., Cao Y. (2017). Layer-by-layer assembly of multilayer thin films for organic optoelectronic devices. Small Methods.

