# Supplementary material for: Polyelectrolyte-Stabilised Magnetic-Plasmonic Nanocomposites
Source: Nanomaterials (Basel). 2018 Dec 13;8(12):1044. doi: 10.3390/nano8121044 (PMC6316294; doi:10.3390/nano8121044)
Supplement: Supplementary file 1 [file nanomaterials-08-01044-s001.pdf]

Supplementary Information

Polyelectrolyte-Stabilised Magnetic-Plasmonic Nanocomposites

Shelley Stafford [1], Coralie Garnier [2] and Yurii K. Gun'ko [1,3*]

1 School of Chemistry, Trinity College Dublin, Dublin 2, Ireland; sstaffo@tcd.ie
2 Institute of Chemistry of Clermont-Ferrand, Sigma Clermont,63170 Aubiere, France; coralie.garnier@sigma-clermont.fr
3 Information Optical Technology Centre, ITMO University, 197101 Saint Petersburg, Russia;

* Correspondence: igounko@tcd.ie; Tel.: +353-1-896-3543

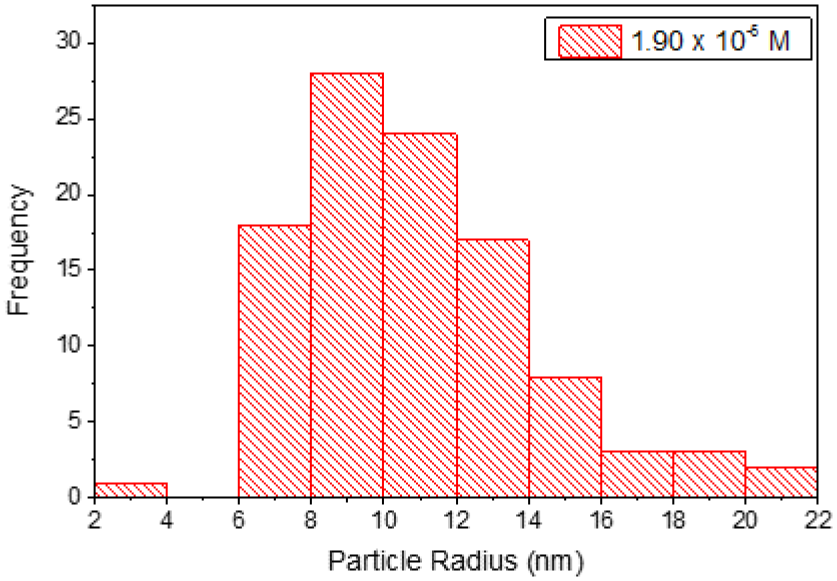

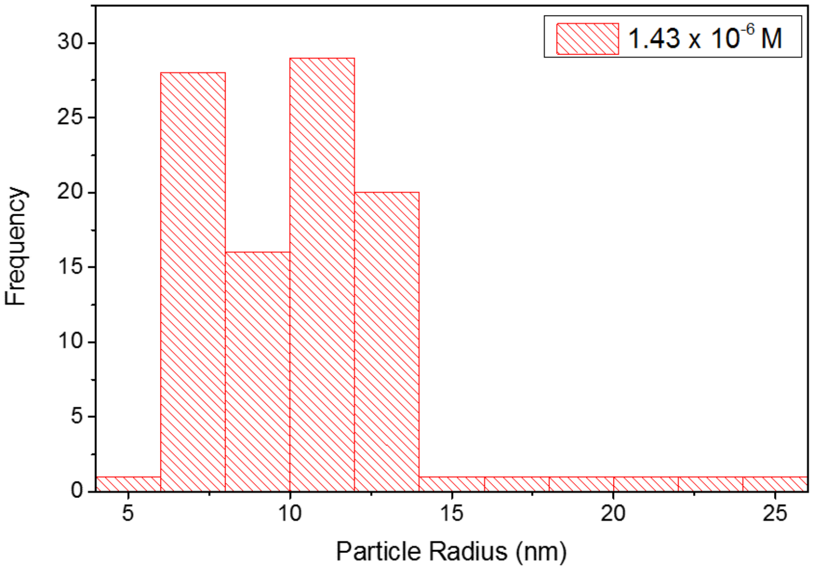

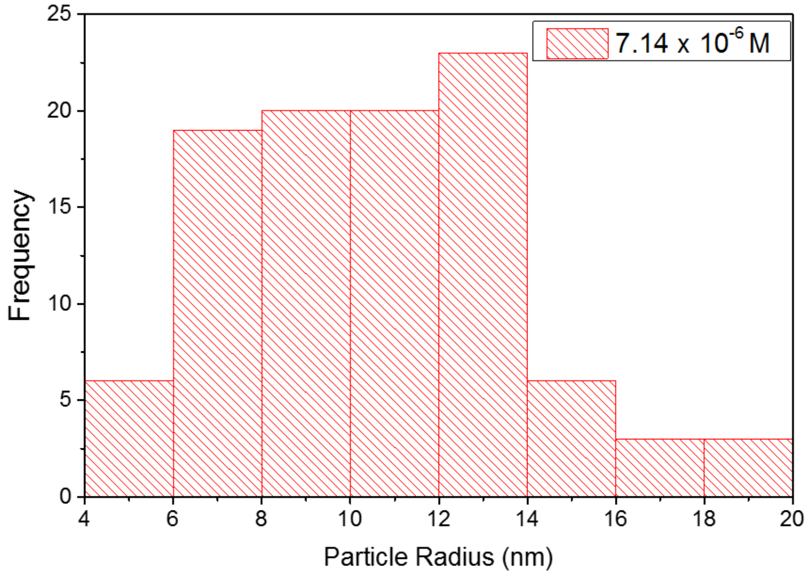

Figure S1. Size Distribution Analysis for PSS-Stabilised Magnetite.

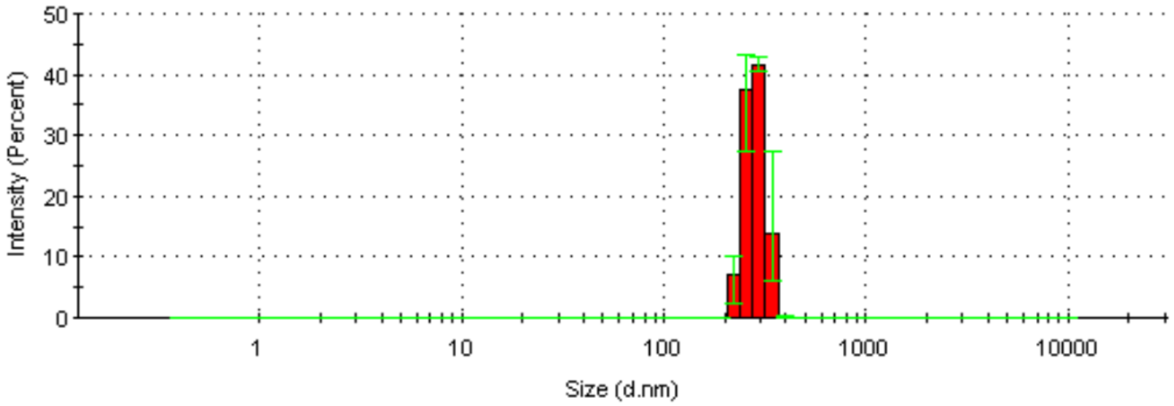

Figure S2. DLS Analysis of PSS-stabilised Fe_3O_4 Nanoparticles.

DLS analysis of PSS-stabilised Fe_3O_4 nanoparticles shows the presence of large aggregates between 200 and 500 nm. This is likely due to the large hydrodynamic radius of the nanoparticles caused by the swelling of the hydrophilic polyelectrolyte. It is also likely due to the fact that multiple nanoparticles may also be suspended across the same long strand of PSS, which will cause large aggregates in solution.

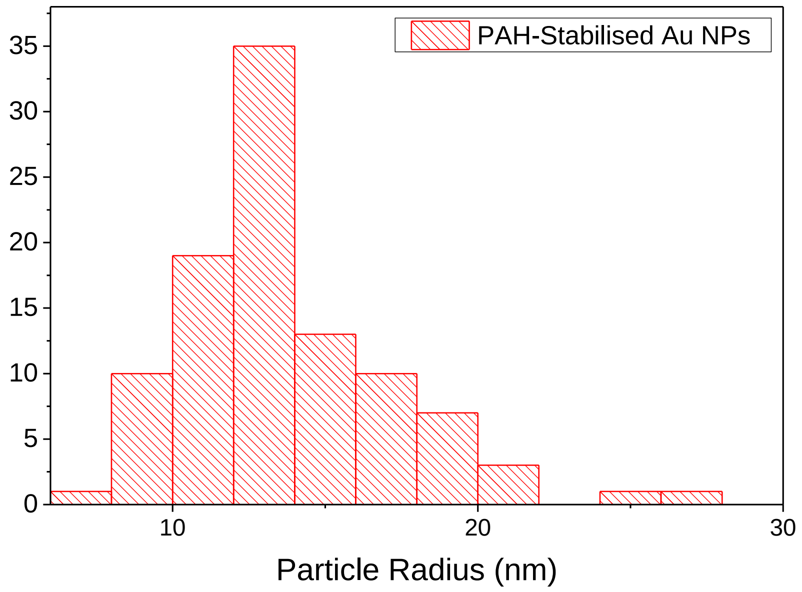

Figure S3. Size Distribution Analysis for PAH-Stabilised Gold Nanoparticles.

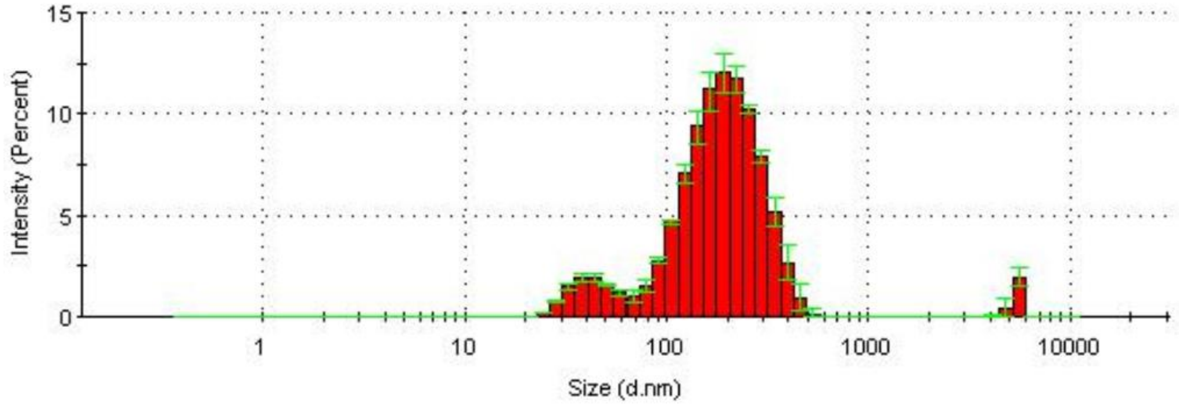

Figure S4. DLS Analysis of PAH-stabilised Au Nanoparticles.

DLS analysis of PAH-stabilised gold nanoparticles show similarly large aggregates spanning from 50 nm to 600 nm in diameter. Similarly to the case for the Fe_3O_4 nanoparticles, this is caused by the large hydrodynamic radius of the PAH and the crosslinking of different polymer chains.

			Mean (mV)	Area (%)	St Dev (mV)
Zeta Potential (mV):	-17.2	Peak 1:	-17.2	100.0	16.9
Zeta Deviation (mV):	16.9	Peak 2:	0.00	0.0	0.00
Conductivity (mS/cm):	0.00276	Peak 3:	0.00	0.0	0.00

Result quality : Good

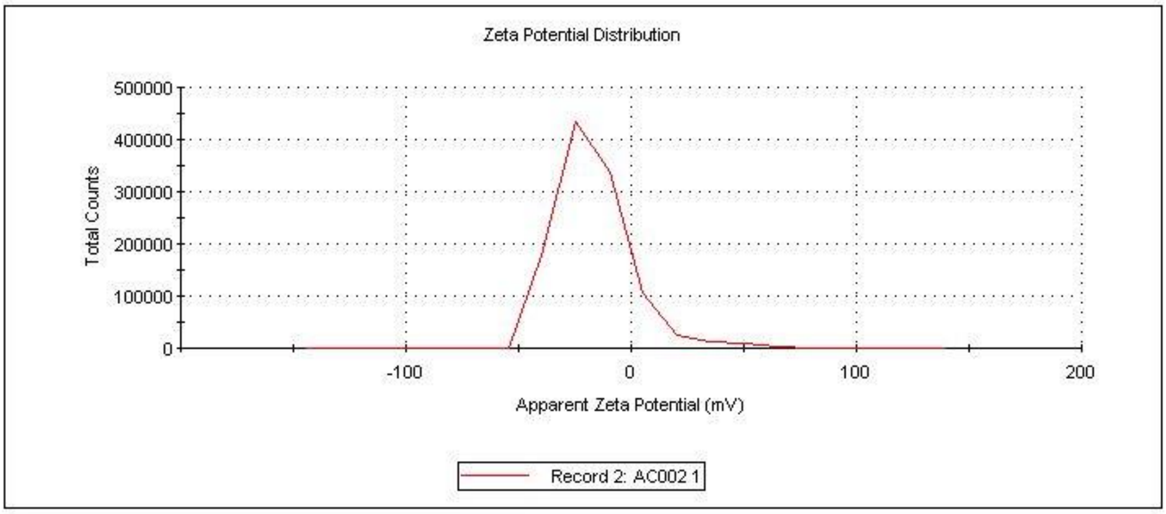

Figure S5. Zeta potential measurements for PSS-stabilised Fe_3O_4 nanoparticles.

			Mean (mV)	Area (%)	St Dev (mV)
Zeta Potential (mV):	19.1	Peak 1:	19.1	100.0	12.7
Zeta Deviation (mV):	12.7	Peak 2:	0.00	0.0	0.00
Conductivity (mS/cm):	0.00268	Peak 3:	0.00	0.0	0.00

Result quality : Good

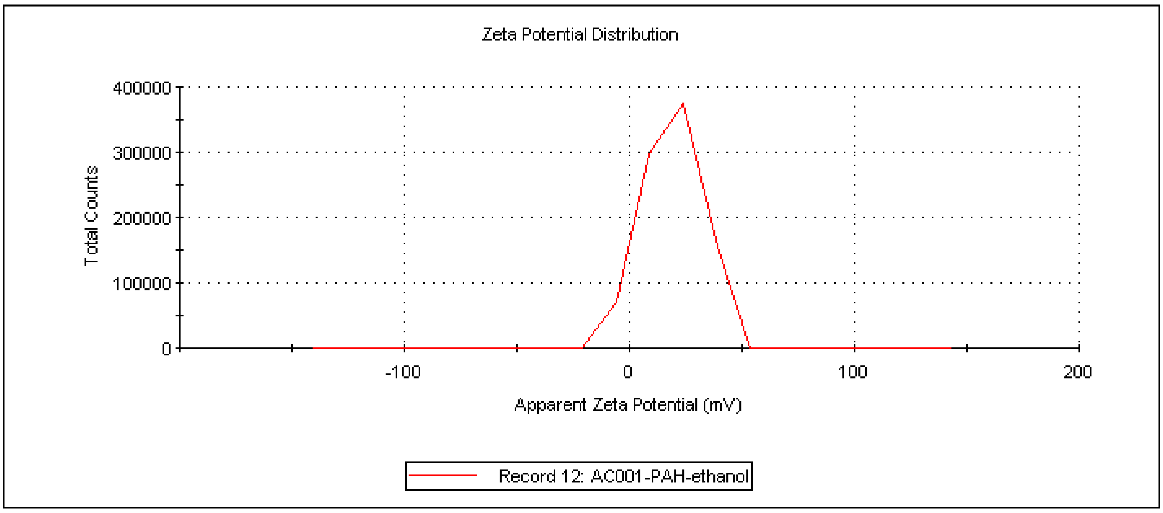

Figure S6. Zeta potential measurements for PAH-stabilised gold nanoparticles.
